# The Effect of Soft Tissue Release at the Thoracolumbar Junction in a Patient with Bilateral Leg Symptoms: A Case Report

**DOI:** 10.31661/gmj.v13i.3357

**Published:** 2024-03-09

**Authors:** Hossein Rafsanjani Deh Qazi, Majid Shahbazi

**Affiliations:** ^1^ Department of Physical Therapy, School of Paramedical and Rehabilitation Sciences, Mashhad University of Medical Sciences, Mashhad, Iran

**Keywords:** Sympathetic Nervous System, Legs, horacolumbar, Manual Therapy, Myofascial Trigger Points

## Abstract

Background: Manual therapists mostly see patients with bilateral leg symptoms. Pain, pins and needles, fatigue, heaviness, lower limb coldness, and loss of neurological conduction are the patients’ symptoms. It is hypothesized to be caused by the sympathetic nervous system. Few publications cover its pathophysiology, diagnosis, and treatment. Limited research has examined the consequences of soft tissue release (STR) at the thoracolumbar junction. This case describes STR in a patient with bilateral leg symptoms.Case Report: A 39-year-old female presented with bilateral leg symptoms, especially the left leg, with more intensity at night. The symptoms started without a clear cause almost two years ago. She had clear low back pain 2 years ago. The sacroiliac joint and neurologic tests were normal. The examiner found some stiffness in the hip joint range of motion and SLR, especially on the left side. The patient reported some stiffness during active lumbar ROM, especially in rotation to the right and flexion. Palpation revealed tenderness in the piriformis, biceps femoris, and gastrocnemius muscles and L5, especially on the left side, and hypomobility in the thoracolumbar junction. The patient has been treated for 10 sessions with a 6-week multimodal approach consisting of STR, the Garston technique, and electrical stimulation in the thoracolumbar junction. The patient was assessed four times. She had a significant decrement in the Numerical Pain Rating Scale (NPRS), the Oswestry Disability Index (ODI), the Global Rating of Change Scales (GRC), and the Beck Anxiety Index following the interventions. She could do her personal activities and would sleep without the sedative.Conclusion: T10 to L2 supply lower extremity sympathetic nerve fibers. This case study demonstrates that these treatments could help these clinical presentations. Interventions with STR need more research. Therapists should evaluate the thoracolumbar junction and SNS in individuals with bilateral leg symptoms without a dermatomal pattern.

## Introduction

**Figure-1:**
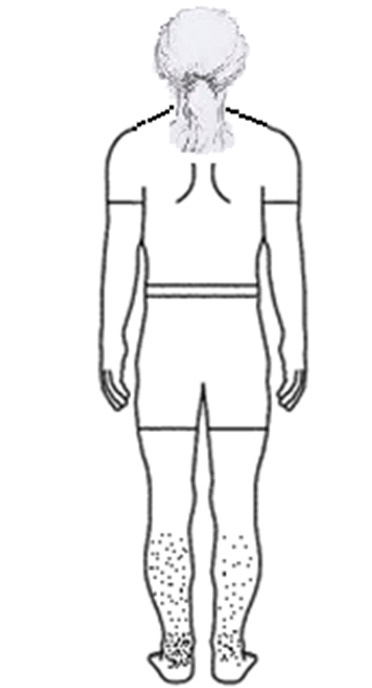
Figure 1. Pain pattern of patient at night

A number of musculoskeletal patients with bilateral lower limb pain refer to medical centers, including physiotherapy [1]. Pain, neurological impairments, fatigue, heaviness sensations, pins and needles, and lower limb coldness are some of the clinical characteristics of their symptoms [1][2]. Clinicians sometimes overlook or misdiagnose symptoms coming from the lower thoracic spine as being lumbar in nature, which causes patients to either not heal at all or recover only partially [1]. The potential pathophysiological pathways creating bilateral leg (BLE) symptoms as a result of thoracic spine involvement are not well understood [1]. The sympathetic nervous system in the T10 to L2 vertebrae innervates the lower extremities [3]. The patients with these symptoms gave a suspicion of a sympathetic system outflowing impairment in the bilateral legs [2]. Similar clinical presentations are reported in patients with T4 syndrome, whose origin is also reported to be a disorder of the sympathetic system [4]. Studies have examined into how injections of paraspinal and epidural anesthetics, as well as thoracic spine joint mobility or manipulation, affect the sympathetic nervous system (SNS) in both patients and healthy individuals [2][5][6][7], but fewer studies have investigated the effects of soft tissue release (STR) of the thoracolumbar junction. This case report covers a patient with symptoms suggestive of SNS dysfunction in the lower extremities, as well as the effects of STR on a patient with bilateral leg complaints.

## Cases Study

**Figure-2:**
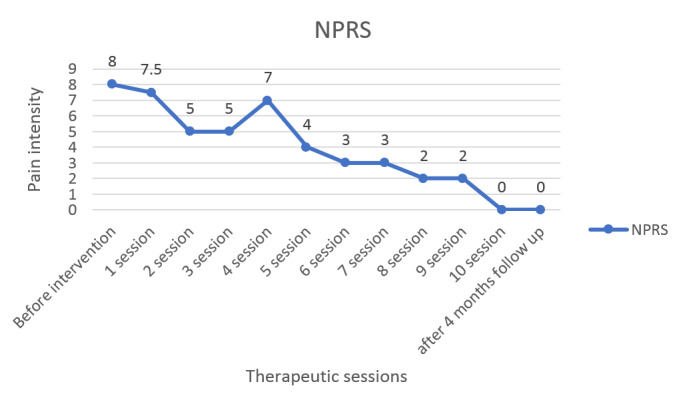
Figure 2. The diagram shows the pain intensity changes during the treatment process and its follow-up.

**Figure-3:**
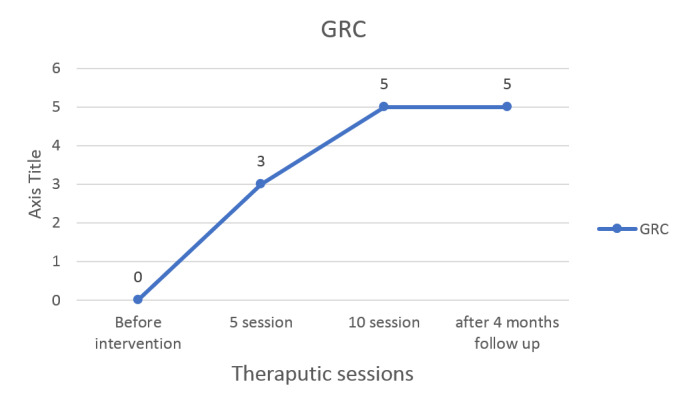
Figure 3. The diagram shows the Global Rating of Change scores during the treatment process and its follow-up.

**Figure-4:**
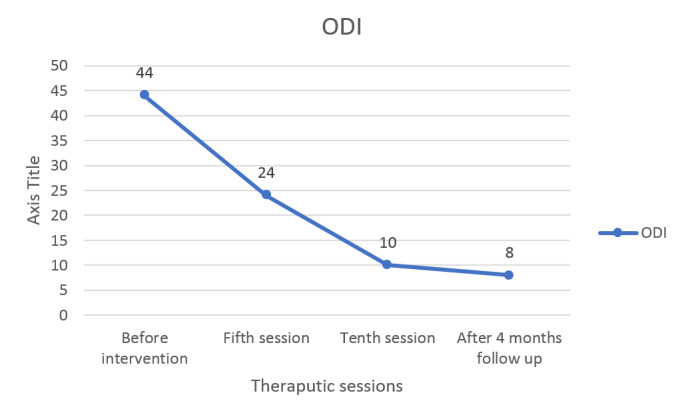
Figure 4. The diagram shows the Oswestry Disability Index changes during the treatment process and its follow-up.

**Figure-5:**
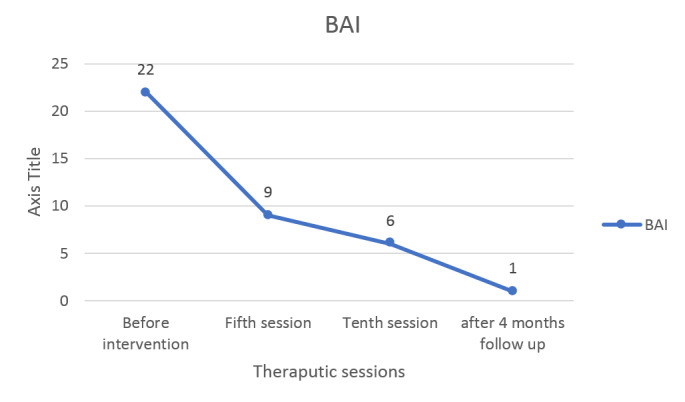
Figure 5. The diagram shows the bake Anxiety Inventory changes during the treatment process and its follow-up.

Subjective Examination

A 39-year-old female presented with bilateral leg symptoms, especially the left leg, with more intensity at night. She reported that her left ankle had more pain, a heavy feeling, and tiredness (Figure-1). The symptoms started without a clear cause almost two years ago. She did have clear low back pain two years ago. She reported some coldness in her legs at night. Aggravating factors were prolonged standing (more than 15-20 min), a lot of physical activities like house cleaning, coldness, and emotional stress. She didn’t have obvious pain during the day, but the severity of the patient's pain was such that it prevented sleep at night, especially when the aggravating factors mentioned above happened to her during the day. She didn’t have a specific medical condition like pelvic inflammatory disease or take a specific drug. The imaging of the spine and lower limb and the lab findings were normal. During the night, to decrease the pain, she took the acetaminophen tablet and tied the legs with a bandage.

Physical Examination

The postural assessment shows some increased lumbar lordosis. During active trunk movement in a standing position, she reported more stiffness on the left side of the trunk and lower limb, but the range of motion of the trunk and hip joints was full. The patient reported stiffness during trunk rotation to the right (VAS =4), forward bending (VAS =3), and extension (VAS =2). Side bending was normal. The sacroiliac joint tests, including the compression test, distraction test, sacral test, and Gaenslen test, were all normal, but during the Patrick test, she complained of pain in the back of her thigh. The neurologic tests were also normal. The examiner found some stiffness in the hip joints' range of motion and SLR, especially on the left side. Palpation revealed tenderness in the piriformis, biceps femoris, and gastrocnemius muscles, and joint play tests on L5 and the thoracolumbar junction showed tenderness. There was some hypomobility in the thoracolumbar junction, especially on the left side.

Outcome Measures

Data on outcome measures was collected prior to intervention, after the fifth and tenth sessions of intervention, and after a four-month follow-up. But for pain intensity, especially at night, evaluation was done every session. The study's key outcomes were the patient's self-reported pain, as evaluated by the numeric pain rating scale (NPRS), and disability, as measured by the Oswestry Disability Index (ODI), as well as improvement changes, as measured by the Global Rating of Change Scales (GRC) [8], and anxiety, measured by the Beck Anxiety Inventory (BAI). The researcher, who was blinded to the intervention, recorded trunk kinematics as one of the secondary outcomes of this study. For measuring the kinematic, the goniometer and tape measure were used.

Treatment

On the basis of the clinical reasoning outlined above, a multimodal approach to treating the condition was utilized. Each of the ten sessions lasted about 70 minutes, including 5 minutes of pre-treatment evaluation, 60 minutes of multimodal approach treatment, and 5 minutes of post-treatment evaluation. A 10-session treatment plan was administered over a six-week period. Each treatment component of the session consisted of a combination of at least 20 min of electrical stimulation with infra-red, 15-30 min of clinical massage, (5-10 min of Garston technique (GT4) over the thoracolumbar junction and quadratus lumborum, and finally 6 min of active stretching of the hamstring and paravertebral muscles. This intervention was conducted by the physiotherapist. After being given sufficient information and signing informed consent forms, the patient made the decision to take part. She was free to choose, at any time, to no longer participate in the study willingly.

Details of Interventions

Details of treatment measures included burst TENS with low-frequency current (1-4 Hz) [9][10] with infra-red heat for at least 20 min on the thoracolumbar junction; STR, including manual stroking, kneading, rolling, friction, and elongation for 15-30 min. After the fourth session, ischemic digital pressure for a myofascial trigger point release of the left quadratus lumborum was added to the treatment program. The Garston technique with GT4 was applied in a direction parallel to the muscle fibers of the paraspinal and quadratum lumborum at a 45° angle. This is quickly followed by 45°-angled muscle therapy along a path perpendicular to the contractile tissue fibers, for a duration of around 5-10 minutes for the entire treatment. The Graston technique included sweeping and fanning movements [11][12]. Finally, for stretching of the hamstring, the patient lay on her back holding her knee from behind, pulling toward the chest, gently straightening the leg at the end position, held for 10 seconds, and repeated 3 sets. For paravertebral muscles, she lay on her back and lifted the involved leg to her chest, then grasped the knee with the opposite hand. She grasped her lower calf with her other hand and gently pulled her leg to the chest [13][14].

## Results

Following 6 weeks of physiotherapy and 4 months of follow-up, the patient exhibited an improvement in the main outcome measures. The NPRS (Figure-2) demonstrated an improvement in the intensity of the pain experienced at night, as well as in feelings of fatigue and heaviness. The GRC scale also showed improvement (Figure-3). The results of the ODI supported a reduction in disability (Table-1) and (Figure-4). Also, the anxiety of the patient improved (Figure-5). Hypomobility of the thoracolumbar junction was im

The secondary outcomes for this study demonstrated a change in lumbar kinematics during trunk movement following the intervention. Following the completion of the final treatment session and a four-month follow-up, the patient reported a complete improvement in the stretching sensation during the movements of the trunk mentioned above.

## Discussion

**Table:** Table1. Details of ODI in a Patient with Bilateral Leg Symptoms Treated with Soft Tissue Release Technique

ODI item	Before intervention	Fifth session	Tenth session	After 4 months follow up
Pain intensity	6	4	0	0
Personal care (washing, dressing etc.)	6	4	0	0
Lifting	8	6	4	2
Walking	0	0	0	0
Sitting	6	2	0	2
Standing	2	2	2	2
Sleeping	6	4	2	2
Sex life (if applicable)	2	0	0	0
Social life	4	2	2	0
Travelling	4	0	0	0
total	38	24	10	8

The intervention at the thoracolumbar junction significantly improved bilateral leg symptoms in this case. There was a dramatic reduction in pain in both legs and ankles at night and an increase in functional ability after 10 sessions of treatments. The results of this case study support a correlation between soft tissue release at the thoracolumbar junction and alleviation of leg symptoms that arouse suspicions of dysfunctional sympathetic outflow. The author knows that it is possible that the symptom relief was not only due to the soft tissue release but that ES with superficial heat can also have an effect on symptoms.

The findings are consistent with previous reports linking bilateral upper and lower extremity symptoms to spinal manual therapy and Kinesio taping [2][3][4][15][16][17]. Some authors demonstrated, in contrast to these results, that manipulation did not cause an instantaneous change in sympathetic function [18]. In manual therapy, myofascial techniques (MFTs) are frequently employed and are thought to lessen pain and stiffness in the tissues. There is, however, little proof of these consequences [18]. There are a variety of reasons that could explain the outcomes of this study. Based on the patient's symptoms, it seems that the patient's problems were due to dysfunction in the sympathetic system [2]. Restoring a tight structure to its natural length is the purpose of soft tissue release therapy (STR), which seeks to reduce discomfort and enhance function [19]. It is stated that impairment in the lower thoracic regions and sympathetic nervous system may cause lumbar spine and leg problems. Consequently, manual therapies of the thoracic area may reduce fascial tension by diminishing sympathetic dominance and aid in pain relief [19], and for the biomechanical effects, Mechanical pressure alters the density, tonus, viscosity, and organization of fascia, suggesting that MFR may result in fascia modification. Another reason that could explain the results is that MFR and ES can stimulate the Ruffini corpuscles (mechanosensitive nerves). Because fascia has a high density of sympathetic nerve terminals, it has been associated with a decrease in the functioning of the sympathetic nervous system of the autonomic nervous system (ANS). Furthermore, activation of the anterior lobe of the hypothalamus has been linked to stimulation of the sensory mechanoreceptors. This results in a general decrease in autonomic muscular activity and mental excitement as well as a change in local tissue viscosity [20]. For more information about the obtained results, there are some studies that showed the effect of back manual therapy on the reduction of anxiety [21][22]. It is shown that long-term chronic pain develops anxiety and depression [23], so it is possible that a decrease in patient anxiety following the back STR decreases the over activity of the sympathetic system. In manual therapy, myofascial techniques (MFTs) are frequently employed and are thought to lessen pain and stiffness in the tissues. There is, however, little proof of these consequences [24]. Restoring a tight structure to its natural length is the purpose of soft tissue release therapy (STR), which seeks to reduce discomfort and enhance function [25].

The treatment approach in this study included multiple components, so the effects of each treatment were unclear. The primary constraints of a case report pertain to the restricted ability to extrapolate the validity of the research and the impracticality of determining a causal relationship.

## Conclusion

This case report demonstrates that 10 sessions of STR over 6 weeks on the paraspinal and quadratus lumborum muscles were effective in reducing bilateral leg symptoms and improving physical function. Also, the anxiety of the patient significantly improved. This study's findings suggest that clinicians should explore the thoracolumbar junction and SNS as part of their treatment plans in patients with bilateral leg symptoms without the dermatomal pattern. It is possible the patient's problem in this study is known as T10 syndrome. Given a lack of published information on this condition, future research on T10 syndrome is recommended.

## Acknowledgement

The patient is appreciated by the authors.

## Conflict of Interest

There were no disclosed conflicts of interest by the writers.
